# Research progress on the mechanisms of neurosyphilis’ impact on cognitive function, early identification, and prevention strategies

**DOI:** 10.3389/fneur.2025.1689945

**Published:** 2026-01-12

**Authors:** Xuechun Lin, Yuying Zheng, Yutang Feng, Xuqi Ren, Meng Cong

**Affiliations:** 1 Department of Dermatology, Affiliated Hospital of Nantong University, Medical School of Nantong University, Nantong, China; 2 Key Laboratory of Neuroregeneration of Jiangsu and Ministry of Education, Co-Innovation Center of Neuroregeneration, Nantong University, Nantong, Jiangsu, China

**Keywords:** cognitive impairment, early diagnosis, neurosyphilis, prevention strategies, *Treponema pallidum*

## Abstract

Neurosyphilis, a severe and often neglected complication of *Treponema pallidum* infection, poses a significant global public health threat, particularly impacting marginalized populations such as men who have sex with men and people with human immunodeficiency virus. Despite global efforts to control syphilis, neurosyphilis is increasingly prevalent, with documented surges of >700% in some regions. Its role in driving cognitive decline often misdiagnosed as psychiatric or neurodegenerative disorders exemplifies “neglected” dimension of diseases. This narrative review synthesizes recent advances in understanding its role in cognitive impairment, spanning from mild deficits to dementia. We detail the multifaceted pathophysiology, including blood–brain barrier disruption, neuroinflammation, abnormal protein aggregation, cerebral small vessel disease, and metabolic dysfunction, which collectively drive neurodegeneration. Critically, we highlight challenges in early diagnosis due to non-specific symptoms and limitations of traditional tests and promising solutions are presented. We also explore novel therapeutic targets and essential public health measures. This comprehensive discussion aims to enhance our understanding the role of neurosyphilis in driving cognitive impairment and potentially contribute to the development of more effective prevention and management strategies.

## 1 Introduction

Neurosyphilis, a systemic complication resulting from *Treponema pallidum* invasion of the central nervous system, remains a significant public health concern globally. Epidemiological data indicate a substantial rise in neurosyphilis incidence worldwide. The centers for disease control and prevention documented a 711% increase in primary and secondary syphilis cases among females and a 174% increase among males in 2021 compared to 2011 within the United States. This rise is particularly pronounced among men who have sex with men (MSM) and people with human immunodeficiency virus (HIV), exacerbating the disease burden of neurosyphilis (1). In China, a region with high syphilis prevalence, the incidence has steadily increased since 2000, with neurosyphilis prevalence reaching 2.1 per 100,000 in certain areas (2).

Neurosyphilis can result in extensive cognitive impairment, with mechanisms involving blood–brain barrier (BBB) disruption, neuroinflammatory responses, and neuronal damage. The clinical manifestations of neurosyphilis are diverse; approximately 66% of neurosyphilis patients exhibit multi-domain cognitive deficits, including impairments in memory, executive function, and visuospatial processing, often misdiagnosed at initial presentation as mental disorders, stroke, cognitive impairment (3, 4). Approximately 40% of syphilis patients with no standardized treatment experience central nervous system involvement, with 59.9% presenting as asymptomatic infections, leading to a high rate of missed diagnoses (3, 4). Therefore, in-depth exploration of the pathological mechanisms of neurosyphilis and the development of early identification and screening techniques with higher diagnostic efficacy are urgently needed.

This narrative review aims to synthesize the molecular mechanisms underlying cognitive impairment in neurosyphilis, along with advances in early identification and preventive measures. The goal is to provide a theoretical basis for optimizing early intervention strategies, thereby improving early diagnosis rates and cognitive outcomes in patients with neurosyphilis.

## 2 Methods

To prepare a narrative review on the neurosyphilis’ impact on cognitive function, we adopted the following methods. Inclusion criteria: Articles published between 2020 and 2025 focusing on the pathophysiological mechanisms, clinical manifestations, early identification, diagnosis, treatment, and prevention of neurosyphilis-induced cognitive impairment; researches involving human subjects or well-validated animal models; original research, meta-analyses, and systematic reviews with rigorous methodological design. Exclusion criteria: Case reports with insufficient sample size (n < 5), non-English literature, articles focusing only on syphilis without involving neuroscience or cognition, and studies with incomplete or unverified data. We searched PubMed^1^ and Web of Science^2^, using advanced search strategies with the combination of MeSH terms and free-text keywords: (“neurosyphilis” OR “neurotreponematosis”) AND (“cognitive dysfunction” OR “cognitive impairment” OR “dementia” OR “mild cognitive impairment”). Subsequently, two independent authors conducted title and abstract screening, followed by full-text evaluation. Disagreements were resolved through discussion with the corresponding authors. We also performed citation tracking of included articles and relevant reviews to ensure no key literature was missed. The research that fits the theme is listed in Table 1.

**Table 1:** Research that fits the theme.

Theme	Chapter	References
Pathophysiology mechanisms	BBB disruption	(5–9)
	Neuroinflammatory response	(6, 10, 11)
	Abnormal protein aggregation	(6, 12, 13)
	Cerebral small vessel disease	(6, 7, 10, 14, 15)
	Metabolic disorder	(4, 10, 16)
Early detection strategies and technological innovations	Optimization of laboratory diagnostics	(3, 13, 32–36)
	Advances in imaging science	(14, 37)
	Artificial intelligence and digital-assisted diagnostics	(28, 38–40)
Early prevention strategies and intervention measures	Screening and risk stratification of high-risk populations	(1, 31, 41–44)
	Standardized antimicrobial therapy and personalized adjustment	(44–47)
	Post-treatment monitoring and recurrence prevention	(41, 44, 48)
	Novel therapeutic targets and immunomodulation	(46, 49, 50).
	Public health administrative measures and health education	(1, 2, 51–60)
Future research directions and clinical translation	An in-depth investigation into the mechanisms of cognitive impairment	(5–7, 10, 46, 61)
	Innovation in precision diagnostic technologies	(13, 28, 62, 63)
	Strategies for treatment optimization and cognitive function preservation	(7, 16, 49, 64)
	Clinical translation and interdisciplinary integration	(14, 38, 40, 65, 66)

## 3 The pathophysiology mechanisms underlying cognitive impairment in neurosyphilis

**Figure 1:**
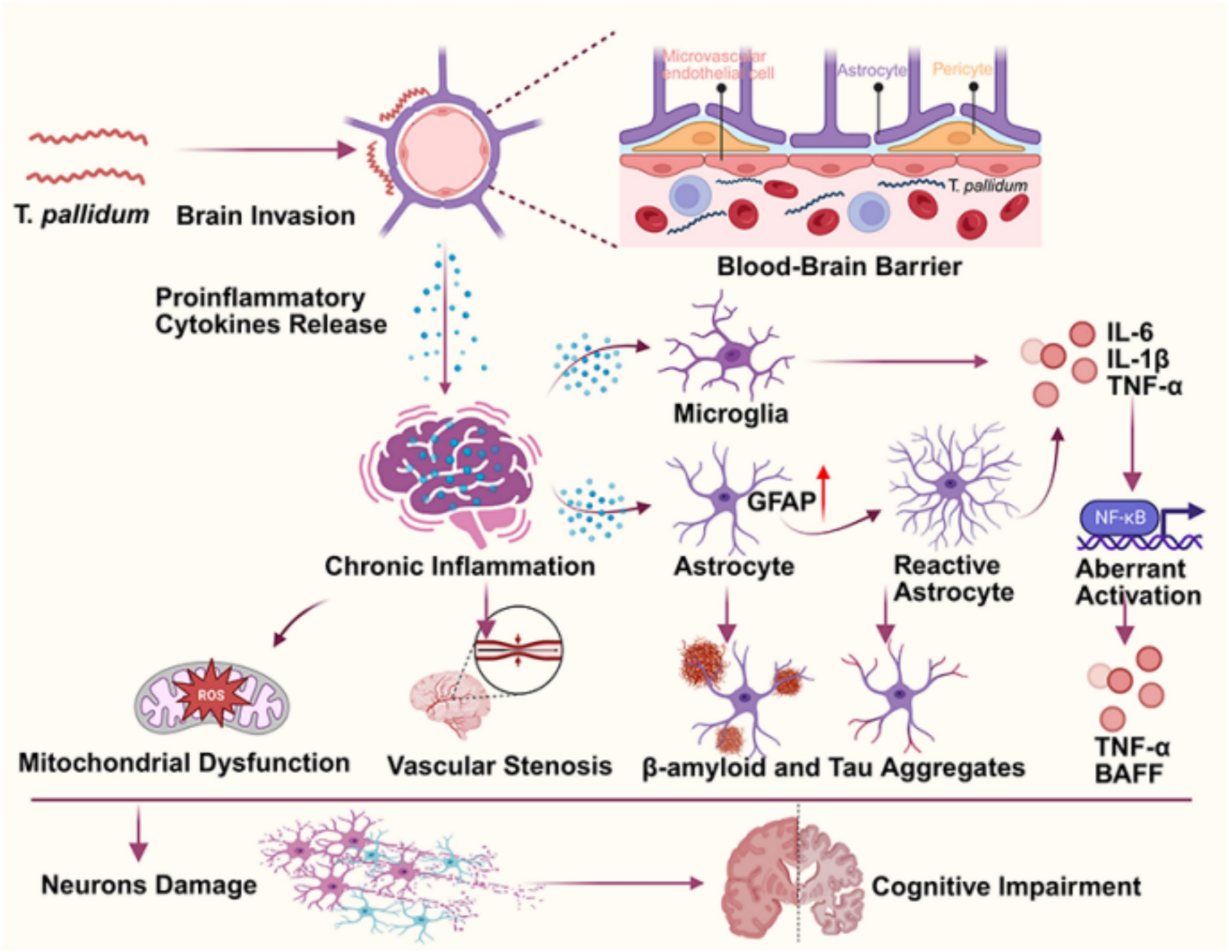
The pathophysiology mechanisms underlying cognitive impairment in neurosyphilis. The figure was created in BioRender.com.

The pathophysiology of neurosyphilis leading to cognitive impairment is a multifaceted process involving direct bacterial invasion, blood–brain barrier disruption, chronic inflammation, neuroinflammation activation, abnormal protein deposition, and metabolic disturbances (Figure 1).

### 3.1 BBB disruption

The BBB comprises endothelial cells, pericytes, astrocytes, and the basement membrane, which collectively regulate substance exchange to maintain brain homeostasis (5–7). *T. pallidum* infection can across the cytoplasmic membrane activate microglia, leading to the release of pro-inflammatory cytokines such as IL-1β and TNF-*α*, subsequently activating the NF-κB signaling pathway. This significantly inhibits the transcriptional expression of tight junction proteins like Claudin-5, ultimately disrupting the BBB structure (6–8). Infection-induced oxidative stress can trigger the abnormal accumulation of reactive oxygen species, attacking pericyte mitochondria and inducing apoptosis. Pericyte reduction or signaling pathway abnormalities lead to BBB damage, reducing cerebral blood flow and causing metabolic waste accumulation, thereby accelerating neurodegeneration and cognitive decline (7). Animal models have confirmed a markedly robust inverse correlation between increased BBB permeability and cognitive function indicators (novel object recognition test, NOR scores), providing evidence for the causal relationship between BBB disruption and cognitive impairment (9).

### 3.2 Neuroinflammatory response

*Treponema pallidum* infection triggers an innate immune response, activating the toll like receptors (TLR) signaling pathway and prompting microglia and astrocytes to release pro-inflammatory cytokines such as IL-6, IL-1β, and TNF-*α* (6, 10). Single-cell transcriptomic analysis reveals a significant increase in the proportion of monocyte-derived macrophages in the cerebrospinal fluid (CSF) of neurosyphilis patients, along with aberrant activation of the NF-κB pathway (10). In the brain tissue of neurosyphilis patients, the TLR4 and cluster of differentiation 14 is significantly upregulated. Furthermore, *in vitro* brain organoid models confirm that peripheral blood mononuclear cells induce the release of inflammatory factors (e.g., TNF-*α*, BAFF) via the TLR4/NF-κB pathway, leading to neuronal dysfunction (10, 11). Astrocytes respond to *T. pallidum*-induced inflammation, including upregulation of glial fibrillary acidic protein (GFAP) and morphological changes (6). Reactive astrocytes promote neuroinflammation by releasing inflammatory mediators and cytokines, further damaging neurons (6). In summary, abnormally activated immune cells can disrupt the integrity of the BBB to trigger a central neuroinflammatory response and cognitive impairment.

### 3.3 Abnormal protein aggregation

TAR DNA-binding protein 43 (TDP-43) undergoes hyperphosphorylation and cytoplasmic aggregation in various neurodegenerative diseases. These aggregates disrupt RNA processing and induce cellular stress responses, with their resultant toxicity contributing to neuronal dysfunction and cell death, thereby exacerbating neurodegeneration and cognitive decline (6). Neurosyphilis further impairs astrocytic proteostasis, promoting the accumulation of neurotoxic protein aggregates such as beta-amyloid and tau, which impair synaptic plasticity and cognitive function, leading to the progression of dementia (6). Serum neurofilament light chain (NfL), a biomarker of axonal injury, is markedly elevated in patients with neurosyphilis and shows a positive correlation with NfL levels in CSF (12, 13).

### 3.4 Cerebral small vessel disease

In patients with neurosyphilis complicated by ischemic stroke, MRI studies have demonstrated that cerebral small vessel disease (CSVD) burden—characterized by white matter hyperintensities, lacunar infarcts, cerebral microbleeds, and perivascular space enlargement—is closely associated with cognitive decline (14). *T. pallidum* infection can involve small and medium-sized arteries, leading to adventitial inflammatory changes, proliferation of smooth muscle cells in the media and intima, and subsequent vascular stenosis or occlusion, resulting in localized cerebral hypoperfusion and cortical atrophy (6, 14). Elevated levels of vascular endothelial growth factor (VEGF) in the CSF of neurosyphilis patients are significantly correlated with neurodegeneration markers such as NfL, suggesting that VEGF may indirectly influence cognitive function by promoting vascular abnormalities and inflammation (15). Additionally, vasculitis can compromise BBB integrity, facilitating infiltration of peripheral inflammatory mediators and immune cells into the brain parenchyma, thereby exacerbating neuroinflammatory cascades (7, 10).

### 3.5 Metabolic disorder

Proteomic studies reveal that neurosyphilis infection significantly suppresses the expression of key energy metabolism proteins such as mitochondrial adenine nucleotide translocase solute carrier family 25 member 4 (SLC25A4) and glucose transporter solute carrier family 2 member 1 (SLC2A1), disrupting mitochondrial oxidative phosphorylation pathways and resulting in a 45% reduction in adenosine triphosphate (ATP) synthesis efficiency. Additionally, it promotes the accumulation of reactive oxygen species, ultimately leading to DNA oxidative damage and loss of synaptic plasticity (10, 16). Further research indicates that the enhanced glycolytic activity induced by recombinant *T. pallidum* protein TP47 alters macrophage metabolic states, causing local energy metabolism imbalance (4). This metabolic dysregulation activates the NLR family pyrin domain containing 3 (NLRP3) inflammasome signaling axis, leading to the excessive release of pro-inflammatory cytokines such as IL-1β and IL-18, thereby establishing a positive feedback loop of neuroinflammation and metabolic disturbance that accelerates neuronal degenerative processes (4). Collectively, existing evidence suggests that metabolic dysregulation coupled with mitochondrial dysfunction contributes to neuronal network abnormalities, which may underlie cognitive impairment such as memory impairment and executive dysfunction observed in affected patients (10). Overall, the pathophysiology of cognitive impairment caused by neurosyphilis is a multifaceted process, and even the result of multiple processes. Therefore, the clinical manifestations and diagnostic challenges of neurosyphilis induced cognitive impairment bring many challenges.

## 4 Clinical manifestations and diagnostic challenges of neurosyphilitic cognitive impairment

### 4.1 Clinical manifestations of neurosyphilis-related cognitive impairment

Cognitive impairment caused by neurosyphilis encompasses a continuum ranging from mild cognitive impairment (MCI) to severe dementia (Table 2). However, the neurological symptoms of neurosyphilis are not specific. Early neurosyphilis can affect the meninges and central blood vessels, including syphilitic meningitis, meningeal vascular neurosyphilis, and syphilitic gingival tumors, typically manifested as headache, nausea, vomiting, confusion, and neck stiffness. Late stage neurosyphilis affects the spinal cord and brain parenchyma, including general paresis and dorsal epilepsy, manifested as ataxia, memory impairment (17–19). The typical neuropsychological profile is characterized by multi-domain amnestic MCI, primarily presenting with memory deficits accompanied by impairments in language, visuospatial abilities, and executive functions, with memory impairment serving as the core diagnostic marker (20). In advanced stages of the disease, untreated patients may progress to neurosyphilis, a subtype marked by neuropsychiatric deficits, including progressive dementia, memory decline, hallucinations, delusions, disorientation, and personality disintegration (21, 22). These clinical features significantly overlap with those observed in schizophrenia, bipolar disorder, and Alzheimer’s disease, leading to elevated misdiagnosis rates at initial presentation; approximately 53.2% of cases are initially misdiagnosed as psychiatric disorders (31.0%), cerebrovascular disease (15.9%), or cognitive impairment (9.0%) (21, 23). Notably, about 10.6% of symptomatic neurosyphilis patients exhibit a distinctive “candy sign”, characterized by rhythmic involuntary contractions of the lower lip, mandible, and facial muscles that mimic sucking movements, which possesses high specificity in differential diagnosis (2). Some cases are misdiagnosed as behavioral variant frontotemporal dementia due to frontal and temporal lobe atrophy; however, the rapid progression of cognitive decline and the potential for neuropsychological recovery following penicillin therapy serve as distinguishing features (24, 25).

**Table 2:** Clinical classification and features of neurosyphilis.

Clinical classification			Typical pathological sites	Core clinical features	Association with cognitive impairment	References
Early neurosyphilis	Asymptomatic neurosyphilis		CSF	Absence of neurological symptoms or signs, with CSF abnormalities (reactive VDRL and CSF lymphocytosis or elevated protein).	Early stage of potential cognitive impairment, screening and early detection are paramount.	(17, 20, 46)
	Symptomatic neurosyphilis	Syphilitic meningitis	Meninges	Headache, nausea and vomiting, nuchal rigidity, and fever.	Common acute cognitive alterations, such as confusion or delirium.	(17, 46)
		Meningovascular neurosyphilis	Small and medium-sized blood vessels	Focal neurologic deficits, such as hemiplegia, hemisensory disturbance, aphasia, and seizures.	Chronic ischemia can lead to vascular cognitive impairment.	(17, 19, 46)
Late neurosyphilis	General paresis		Cerebral cortex (frontal and temporal lobes).	Progressive dementia, personality changes, psychiatric symptoms (delusions and hallucinations), and neurologic signs(tremor, dysarthria, hyperreflexia, and Argyll Robertson pupils).	A core cognitive phenotype, ranging from mild cognitive impairment to severe dementia.	(18, 20, 21)
	Tabes dorsalis		Dorsal columns and dorsal roots.	Lightning pains, impairment of deep sensation, urinary incontinence, Charcot joints, Argyll Robertson pupils, and visceral crises (episodes of severe pain in sites such as the stomach, larynx, or rectum).	Atypical cognitive impairment, with predominant involvement of spinal sensory pathways, can coexist with General Paresis.	(19)
Gummatous neurosyphilis			A focal granulomatous lesion of the meninges, brain parenchyma, or spinal cord.	It manifests with mass effect, mimicking a brain tumor or abscess, and results in focal neurologic deficits and increased intracranial pressure.	Can lead to cognitive impairment associated with focal neurologic deficits, such as in the domains of language or executive function.	(17)

### 4.2 Diagnostic challenges of neuro-syphilitic cognitive impairment

The diagnosis of neurosyphilis usually requires the integration of epidemiological information, neurological or neuropsychiatric manifestations, serological analysis of blood and CSF, and in specific cases, imaging evaluation. For syphilis patients with neurological symptoms, almost all major guidelines recommend lumbar puncture (26, 27). At present, diagnostic challenges primarily stem from four major issues: firstly, the lack of standardized diagnostic criteria. Significant discrepancies exist among authoritative guidelines regarding CSF testing parameters, with regional variations in testing methodologies based on local resource availability, resulting in an inability to unify laboratory parameters recommended by guidelines (28). Some patients, despite negative CSF venereal disease research laboratory (VDRL) results, may still be diagnosed with neurosyphilis due to CSF abnormalities such as elevated protein levels or increased white blood cell counts, along with clinical manifestations including cognitive impairment (29). Secondly, traditional detection methods exhibit limited sensitivity. Meta-analyses indicate that CSF VDRL specificity can reach up to 98.3%, whereas sensitivity may be as low as 48.9%, with the potential for false negatives influenced by the prozone phenomenon (30). Thirdly, neuroimaging findings lack specificity. Although some patients display medial temporal lobe FLAIR hyperintensities, similar imaging features are also observed in autoimmune encephalitis and herpes simplex virus encephalitis (28). Fourthly, the diagnostic complexity in special populations is notable. Studies reveal that the incidence of neurosyphilis among HIV-infected individuals is substantially higher than in the general population (42 versus 0.3 per 100,000), yet CSF white blood cell counts do not differ significantly between HIV-positive and HIV-negative groups (31). Actually, researchers have also made many efforts, such as improving sample preprocessing methods and seeking inspiration from case reports to discover new sample types. However, there is currently no gold standard for diagnosing neurosyphilis, making it difficult to evaluate the diagnostic efficiency of new methods. In the following text, we summarize the relevant early detection strategies and technological innovations.

## 5 Early detection strategies and technological innovations

### 5.1 Optimization of laboratory diagnostics

Current early diagnostic laboratory systems primarily rely on CSF white blood cell count, protein concentration, and VDRL testing; however, their sensitivity and specificity are limited, and the invasive nature of lumbar puncture restricts patient compliance (32). In recent years, the introduction of inflammation-related biomarkers such as CSF chemokine CXCL13 and IL-10 has significantly improved the detection rate of asymptomatic infections (33). Luo et al. explore the amyloid and tau metabolism in neurosyphilis patients in different stages, the levels of Alzheimer-type biomarkers in general paresis and asymptomatic neurosyphilis patients in comparison to patients with Alzheimer’s disease and normal controls were investigated. Research has demonstrated that general paresis, asymptomatic neurosyphilis patients and Alzheimer’s disease patients are characterized by distinct patterns of the CSF biomarkers Aβ and tau. That is, the CSF Aβ42 level in general paresis patients was significantly lower than that in asymptomatic neurosyphilis patients and the normal control group, but higher than that in AD patients. There was no significant difference in the CSF Aβ42 level between asymptomatic neurosyphilis patients and the normal control group. Therefore, different levels of CSF Aβ could be helpful for the differentiation between different stages of neurosyphilis (34). Different from AD patients, general paresis patients had a intermediately reduced CSF Aβ42 level and normal tau protein level. This suggests that in the late stage of neurosyphilis, there may be a persistent Aβ metabolic disorder related to chronic inflammation in the brain. The authors speculated that the adhesion and invasion to host cells through the surface components of *T. pallidum* can directly affect brain function and Aβ metabolism. On the other hand, the inflammatory response triggered by *T. pallidum* molecules may indirectly interfere with the balance of Aβ production or clearance (34). Neurodegeneration markers in CSF, including NfL, GFAP, and ubiquitin carboxyl-terminal hydrolase L1 (UCH-L1), are closely associated with cognitive decline, and combined testing enhances diagnostic accuracy (13). Serum levels of GFAP and UCH-L1 show significant correlation with CSF concentrations, with sensitivities and specificities for neurosyphilis diagnosis reaching 80.4%/78.4 and 90.2%/92.16%, respectively (13, 35). Neutrophil-related parameters such as the CD64 index and neutrophil-to-lymphocyte ratio are elevated specifically in the peripheral blood of neurosyphilis patients, particularly in cases with negative CSF-TRUST results, demonstrating excellent discriminative value (36). For patients with negative MRI findings, metagenomic next-generation sequencing (mNGS) in CSF can detect low-abundance *T. pallidum*, highlighting its substantial potential for identifying low-level infections (3).

### 5.2 Advances in imaging science

The neuroimaging manifestations of neurosyphilis are highly heterogeneous, encompassing a range of pathological changes such as cerebral infarction, white matter hyperintensities, global brain atrophy, and hippocampal atrophy (37). Studies have confirmed that 42.1% of patients receiving standard treatment exhibit progressive radiological alterations, underscoring the critical importance of dynamic imaging surveillance (37). Distinct imaging features are observed across different clinical subtypes: neurosyphilis is characterized by temporal lobe and hippocampal atrophy, whereas meningeal vascular neurosyphilis predominantly presents with infarcts in the basal ganglia and thalamic regions (37). Patients with neurosyphilis demonstrate a significantly higher burden of CSVD compared to non-infected controls, with this burden correlating with acute cognitive impairment and adverse long-term cognitive outcomes. Quantitative radiomics analysis of white matter hyperintensities, microbleeds, asymptomatic lacunar infarcts, and perivascular spaces provides superior predictive value for cognitive prognosis in neurosyphilis patients compared to single biomarkers (14).

### 5.3 Artificial intelligence and digital-assisted diagnostics

A machine learning model based on extreme gradient boosting effectively differentiates diagnoses by integrating clinical symptoms, CSF parameters (protein, WBC, VDRL), and serum non-treponemal tests, demonstrating stable generalization across regional cohorts (28). In biosensing, AI-enabled wearables facilitate early detection of cognitive and emotional states via continuous electrooculogram and EEG monitoring (38). Multiple systematic reviews and meta-analyses indicate that Montreal Cognitive Assessment (MoCA), Mini-Mental State Examination (MMSE), and Clock Drawing Test (CDT) are the most commonly used cognitive screening tools for MCI, with MoCA showing greater sensitivity to subtle cognitive changes than MMSE (39). The Digital Clock and Recall (DCR) test, a digital cognitive assessment tool combining CDT and word recall tasks, surpasses MMSE in sensitivity, offers shorter testing times, and reduces racial and linguistic biases. DCR’s automated analysis minimizes subjective judgment errors, standardizes testing procedures, and is suitable for widespread adoption in primary healthcare settings (40).

## 6 Early prevention strategies and intervention measures

### 6.1 Screening and risk stratification of high-risk populations

The prevention and control system for neuro-syphilis-related cognitive impairment centers on proactive screening of high-risk populations. Individuals with serum rapid plasma reagin (RPR) titers ≥1:32, HIV-infected patients (particularly those with CD4 + T cell counts ≤350/μL), MSM, and those who have not received standardized treatment are identified as high-risk groups (1, 31, 41). In cases of HIV co-infection, CSF analysis—specifically CSF-VDRL and TPPA—is routinely performed regardless of clinical symptoms upon syphilis diagnosis. Persistent high serum RPR titers or failure to achieve a fourfold decline post-treatment (serofast state) necessitate repeated CSF evaluation (42, 43). Even among persistently high-risk individuals receiving antiretroviral therapy, ongoing monitoring of CSF white blood cell count and protein levels is essential to assess treatment response and the risk of relapse (44).

### 6.2 Standardized antimicrobial therapy and personalized adjustment

Given its reliable CSF penetration, intravenous penicillin G (18–24 million units per day for 10–14 days) remains the first-line treatment option (44, 45). For penicillin-allergic patients, intravenous ceftriaxone (1–2 g per day for 10–14 days) demonstrates comparable bactericidal efficacy in CSF and is effective against both sensitive and potentially resistant strains (46, 47). Doxycycline is also considered an acceptable alternative, particularly for early and late latent syphilis, although further research is needed to establish optimal high-dose regimens for neurosyphilis (45). Currently, there is no evidence that additional antibiotic courses improve long-term clinical outcomes in serofast patients; thus, routine adjunctive therapy is not recommended, and close monitoring for reinfection is advised (45). Individualized treatment adjustments should consider CSF cell counts, serological responses (such as serofast state), and patient allergy or resistance profiles.

### 6.3 Post-treatment monitoring and recurrence prevention

The criteria for successful treatment include normalization of CSF white blood cell count and seroreversion of VDRL. In cases with prior CSF abnormalities, repeat lumbar punctures are recommended at 3, 6, and 12 months post-therapy (44). Regular monitoring of serum RPR titers is essential; failure to achieve a fourfold decline within 6 to 12 months post-treatment suggests persistent serofixation or potential relapse (48). Studies indicate that patients with CSF pleocytosis who receive neurosyphilis treatment have a significantly lower risk of cognitive decline compared to untreated individuals (HR = 0.24, 95% CI 0.07–0.88, *p* = 0.03) (41). Elevated CSF WBC count (>5/μl), serving as a key indicator of the severity of cognitive impairment, should be monitored post-therapy to guide ongoing management and prevent deterioration (41). Systemic inflammation may accelerate cognitive decline; therefore, even high-risk patients not meeting the diagnostic criteria for neurosyphilis—such as HIV-infected individuals with CD4 + counts ≤350/μl or serum RPR titers ≥1:32—should be evaluated for CSF inflammatory markers. Targeted interventions to reduce inflammation are recommended to mitigate long-term cognitive deterioration (41).

### 6.4 Novel therapeutic targets and immunomodulation

*Treponema pallidum* activates the mTORC1 signaling pathway, suppressing TFEB-mediated lysosome biogenesis, which leads to autophagosome accumulation and increased microglial apoptosis, thereby exacerbating neuroinflammation and cognitive impairment. mTORC1 inhibitors targeting this pathway, such as rapamycin, have been shown to reverse these effects (49). Concerning the BBB disruption mechanism, natalizumab (an anti-*α*4 integrin monoclonal antibody) reduces peripheral immune cell infiltration into the central nervous system, decreasing glial activation and levels of pro-inflammatory cytokines such as IL-6 and TNF-α in animal models, which significantly improves cognitive performance in mice (50). Further genetic regulation studies have identified polymorphisms in the IL-10 promoter region associated with the risk of neurosyphilis, particularly the GG genotype at the rs1800896 locus, which correlates with higher IL-10 secretion. Elevated IL-10 levels may mitigate central nervous system inflammation, thereby protecting neuronal function and facilitating cognitive recovery post-treatment (46).

### 6.5 Public health administrative measures and health education

Many countries have implemented mandatory reporting of all newly diagnosed syphilis cases, utilizing integrated data from hospitals, laboratories, and disease control centers to facilitate case tracking. The United States implemented the National Syphilis Elimination Plan in 2021, focusing on targeted screening of high-risk groups (MSM, HIV infected people and sex workers), mandatory reporting of syphilis cases, and incorporating syphilis and HIV testing services into community health centers. The plan has reduced the incidence rate of neurosyphilis among HIV positive people by 15% within 3 years (51, 52). China has also launched the National Plan for the Control of Sexually Transmitted Diseases (2021–2025), emphasizing the hierarchical management of syphilis cases, free screening of high-risk populations in key areas, and training on neurosyphilis diagnosis for grassroots medical personnel. However, regional differences in screening coverage still exist, especially in rural areas (2, 53, 54). The European Union has adopted a “unified monitoring system” among its member states to standardize CSF testing parameters and treatment plans. By establishing cross-border cooperation for monitoring drug-resistant *T. pallidum* strains, a model has been provided for regional coordination (55, 56). These approach aids in early outbreak detection and resource allocation (57). Co-infection with HIV may exacerbate serological non-responsiveness, necessitating more rigorous follow-up and treatment protocols. Establishing a synergistic syphilis-HIV prevention and control system is a critical strategy for the early diagnosis of neurosyphilis (1, 58). Clinical quality assessments reveal that misdiagnosis of neurosyphilis is partly due to healthcare professionals’ insufficient recognition of atypical neurological symptoms, such as band-like sensations and the candy sign, highlighting the urgent need for standardized training programs to enhance clinical diagnostic reasoning (2). Studies indicate that 33.5% of neurosyphilis patients are co-infected with other sexually transmitted infections, such as chlamydia, mycoplasma, and HPV.

The recurrence of syphilis and its severe neurological complications highlights the serious failure and inadequacy of current sexual health strategies in many regions. In order to effectively curb the incidence rate of neurosyphilis, we must change to comprehensive, evidence-based and de stigmatized sex education. Education initiatives must go beyond HIV focused information transmission, clearly including the possibility of serious long-term consequences such as syphilis transmission, subtle early symptoms, cognitive impairment, and the importance of early diagnosis and treatment. These projects should be tailored to key populations such as men who have MSM, adolescents, and individuals with multiple sexual partners through various channels such as schools, community health centers, and digital platforms (59). In addition, syphilis prevention cannot be isolated. It must be embedded in a powerful comprehensive prevention and control framework for sexually transmitted infections. Regular syphilis testing can be promoted as part of standard healthcare, especially for high-risk populations, rather than just for patients with existing symptoms. At the same time, strengthen the integration of syphilis testing with all HIV care institutions, and fully utilize existing healthcare to promote the detection of corresponding cases. It is important to eliminate the stigma associated with sexually transmitted infections and reduce the cost of testing and treatment, which is crucial for improving healthcare seeking behavior. Further strengthening the use of social media and telemedicine for cautious education and notification can significantly expand coverage and effectiveness (60). By combining public health monitoring with targeted and effective sex education and accessible comprehensive clinical services, sustainable defense measures can be established. Overall, strengthening safe sex education and regular sexual health screenings are essential measures (2).

## 7 Future research directions and clinical translation

### 7.1 An in-depth investigation into the mechanisms of cognitive impairment

The pathogenic mechanisms underlying neurocognitive impairments associated with neurosyphilis remain incompletely elucidated. Disruption of the BBB, neuroinflammatory responses, and abnormal protein deposition are likely to collectively contribute to the core pathological processes (5–7, 10). Proteomic studies have confirmed that central nervous system inflammation is primarily mediated by monocyte-derived macrophages via the TLR/NF-κB signaling pathway, a process significantly correlated with neuronal degenerative changes (10). *In vitro* experiments demonstrate that *T. pallidum* infection induces microglial activation, accompanied by abnormal amyloid-beta deposition, suggesting overlapping molecular mechanisms with Alzheimer’s disease (61). Currently, brain organoids lack functional vascular structures, limiting investigations into BBB disruption mechanisms. Future research should focus on developing more precise vascularized brain organoid models, integrating single-cell sequencing techniques to analyze the molecular responses of specific neuronal subpopulations, such as neurons and glial cells. The molecular pathways by which *T. pallidum* evades immune detection and establishes persistent latency within the central nervous system remain to be elucidated through multi-omics approaches, including gene editing and metabolomics (46).

### 7.2 Innovation in precision diagnostic technologies

Current diagnosis of neurosyphilis still relies on CSF analysis; however, its invasive nature limits widespread clinical application. Research into serum biomarkers offers new hope for non-invasive diagnosis, with markers such as NfL, GFAP, and UCH-L1 demonstrating strong correlations with CSF indicators (13). mNGS enables direct detection of *T. pallidum* DNA from CSF, representing a breakthrough in the early identification of asymptomatic neurosyphilis (62). Machine learning-based multi-omics models integrating clinical symptoms, CSF parameters, and serum non-treponemal tests have shown excellent performance in the differential diagnosis of neurosyphilis (28). A molecular detection technique combining CRISPR-Cas13a targeting *T. pallidum* genes (such as tp0574) significantly enhances sensitivity and specificity; future research should explore its application in molecular diagnostics of neurosyphilis (63).

### 7.3 Strategies for treatment optimization and cognitive function preservation

Although penicillin remains the standard therapeutic approach, some patients do not achieve complete recovery of cognitive function post-treatment, indicating the need to explore neuroprotective adjuvant therapies (64). *T. pallidum* induces microglial apoptosis by inhibiting autophagosome-lysosome fusion, while mTORC1 inhibitors restore autophagic flux; however, their clinical application warrants further investigation (49). In animal models, nanocarrier delivery systems facilitate BBB penetration via low-frequency pulsed ultrasound, which modulates the endoplasmic reticulum unfolded protein response to alleviate endoplasmic reticulum stress (16). Therapies aimed at restoring BBB integrity, such as regulating tight junction protein Claudin-5 expression, may represent a novel strategy to prevent cognitive decline (7).

### 7.4 Clinical translation and interdisciplinary integration

Overall, in the future, further research should be conducted on the mechanism of *T. pallidum* induced blood–brain barrier disruption, and the diagnosis of neurosyphilis should be transformed into non-invasive diagnostic tools. The development of vascularized brain organoid models should prioritize clinical applicability, such as simulating the pathological microenvironment of neurosyphilis in HIV co infected patients to test the efficacy of personalized drug combinations. The advancement of neuroimaging genomics has enabled quantitative assessment of small vessel pathologies such as white matter hyperintensities and microbleeds, serving as crucial tools for predicting cognitive prognosis (14). AI-assisted electroencephalography and functional near-infrared spectroscopy facilitate early detection and dynamic monitoring of MCI (38, 65). Developing diagnostic systems based on machine learning algorithms that integrate clinical data, biomarkers, and imaging features can enhance early diagnosis, patient stratification, and prediction of treatment response (40, 66). Future research should focus on multimodal data fusion algorithms, validation of predictive models in clinical trials, and the integration of real-time monitoring data to optimize dynamic therapeutic strategies. Machine learning models that integrate multiple omics data such as CSF biomarkers, imaging features, and clinical parameters should be validated in multicenter clinical trials to ensure their reliability in guiding early diagnosis and treatment selection. In addition, deciphering the *T. pallidum* latency mechanism through microbiology, elucidating the pathways of cognitive impairment through neuroscience, conducting clinical screening and patient management through dermatology and venereology, further developing immunomodulatory therapies through immunology, and optimizing AI assisted diagnosis through data science. At the same time, a multidisciplinary research alliance for neurosyphilis can be further established, integrating resources from basic research institutions, clinical hospitals, and public health institutions to accelerate the translation of mechanism research results into clinical practice. Interdisciplinary efforts based on multiple disciplines are crucial for translating basic research into clinical practice, advancing precise diagnosis, targeted therapy, and vaccine development, while strengthening public health interventions to alleviate the cognitive burden associated with neurosyphilis.

## 8 Conclusion and discussion

**Figure 2:**
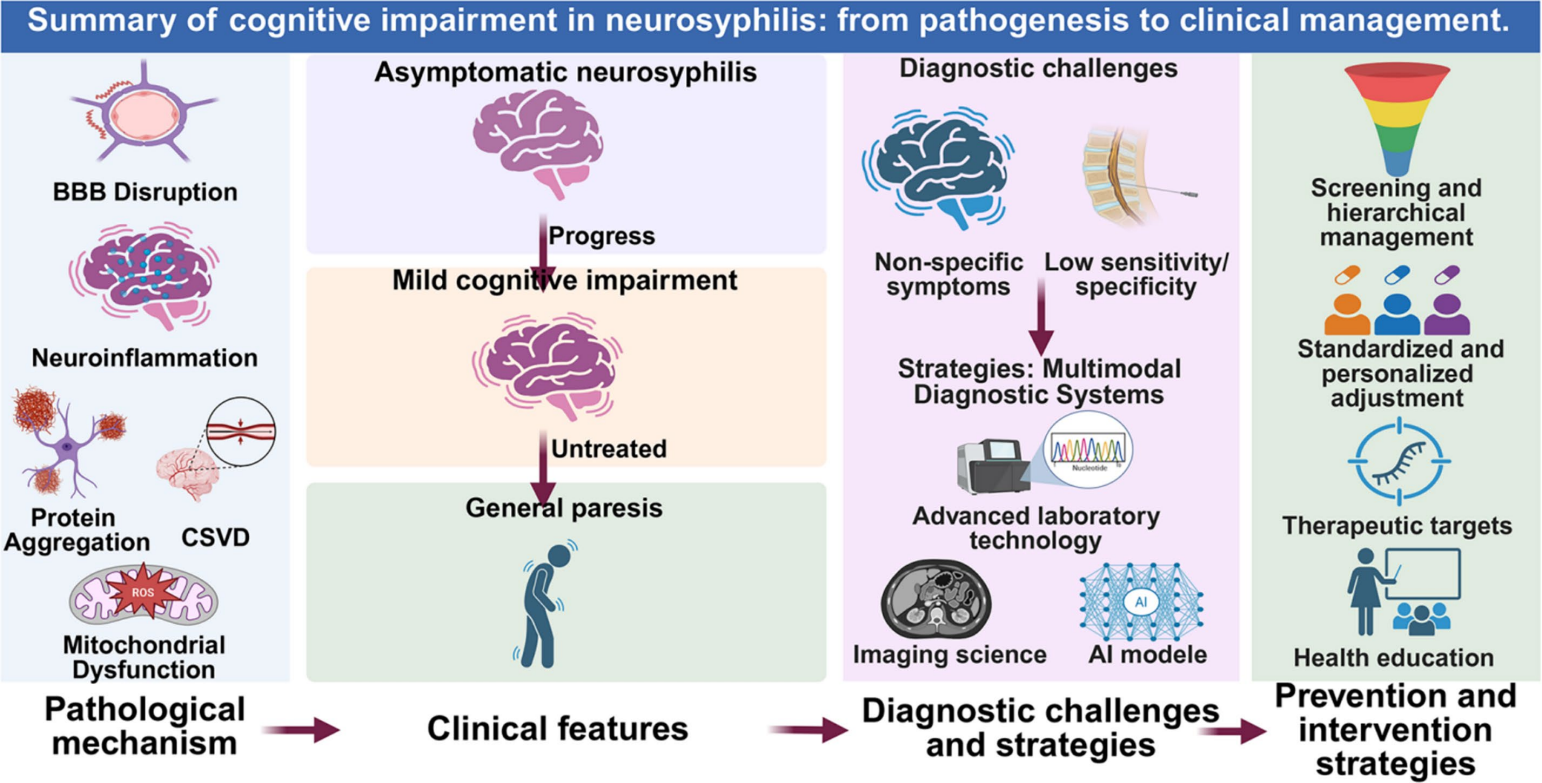
Summary of cognitive impairment in neurosyphilis: from pathogenesis to clinical management. This schematic integrates the key pathophysiological mechanisms, clinical progression, diagnostic challenges, and corresponding intervention strategies discussed in this review. The figure was created in BioRender.com.

In recent years, the global incidence of neurosyphilis has continued to rise, emerging as a significant public health concern. Neurosyphilis induces cognitive impairment through multiple pathogenic mechanisms (Figure 2). A multimodal diagnostic system for early detection of neurocognitive damage has been gradually established: mNGS demonstrates considerable potential in detecting low-level infections (3, 13). Radiomics techniques quantifying CSVD burden facilitate early prognostic prediction of cognitive outcomes, while AI models exhibit excellent performance in differential diagnosis (14, 28). Preventive strategies emphasize risk stratification and management of high-risk populations: routine CSF monitoring or ongoing assessment for individuals with serum RPR titers ≥1:32, HIV-infected patients, and MSM, with penicillin as the first-line treatment, adjusting personalized therapy based on serological response, allergy, or drug resistance (42–45). At the public health level, strengthening syphilis-HIV co-screening networks, enhancing healthcare professionals’ awareness of atypical neurological symptoms, and promoting public education on safe sexual practices are imperative (1, 2, 58) (Figure 2).

Future research should focus on the following directions: at the mechanistic level, utilizing single-cell sequencing to analyze the molecular response characteristics of specific neuronal subpopulations and establishing brain organoid models to investigate the mechanisms of BBB disruption (10); advancing diagnostic techniques through the study and application of serum biomarkers and validating the diagnostic efficacy of machine learning-based multi-omics models in clinical settings (28, 63); optimizing therapeutic strategies by exploring novel pharmacological agents and neuroprotective adjunct therapies rooted in mechanistic insights (7, 16, 49); and in terms of clinical translation, verifying the predictive accuracy of AI-assisted diagnostic systems for cognitive prognosis and developing dynamic treatment protocols based on real-time monitoring data (40, 66). Ultimately, integrating multidisciplinary efforts from microbiology, neuroscience, dermatology and venereology, and data science is essential to establish innovative pathways for the precise prevention and control of neurosyphilis-associated cognitive impairment.

In summary, neurosyphilis is a serious and often overlooked complication of central nervous system infection in syphilis patients. Although neurosyphilis has been discovered for centuries, the mechanism by which neurosyphilis causes cognitive impairment still requires further research. The role of neurosyphilis in causing cognitive decline is often misdiagnosed as a mental illness or neurodegenerative disease. Patients often seek treatment in neurology, psychiatry, or general medicine first due to various neurological symptoms such as headaches, dizziness, cognitive decline, limb weakness, and sensory disorders, rather than dermatology. At present, the diagnostic methods for early detection of neurocognitive impairment are relatively comprehensive, but there is still a long way to go in strengthening sexual education for patients and reducing their shame toward sexually transmitted diseases. Furthermore, it is crucial to enhance the awareness of healthcare professionals toward atypical neurological symptoms. Early serological and cerebrospinal fluid testing can effectively reduce the rates of missed diagnosis and misdiagnosis. Given the sustained sensitivity of *T. pallidum* to penicillin, the prevalence of neurosyphilis implies a neglect of prevention and management, therefore it is necessary to develop new drugs and vaccines. The latest research in molecular biology provides unprecedented opportunities for the development of new drugs and vaccines targeting *T. pallidum*. In the future, further research on the mechanisms of cognitive impact of neurosyphilis needs to be translated into efficient early diagnosis strategies, and the most cost-effective personalized treatment measures need to be developed to alleviate the global burden of neurosyphilis.

## Footnotes

1
https://pubmed.ncbi.nlm.nih.gov

2
https://www.webofscience.com
